# Pretreatment “prognostic nutritional index” as an indicator of outcome in lung cancer patients receiving ICI-based treatment: Systematic review and meta-analysis

**DOI:** 10.1097/MD.0000000000031113

**Published:** 2022-10-28

**Authors:** Yifeng Shao, Wei Cao, Xinliang Gao, Mingbo Tang, Dongshan Zhu, Wei Liu

**Affiliations:** a Department of Thoracic Surgery, The First Hospital of Jilin University, Changchun, Jilin, China; b Department of Urinary Surgery, The First Hospital of Jilin University, Changchun, Jilin, China.

**Keywords:** immune checkpoint inhibitors, lung cancer, overall survival, prognostic nutritional index, progression-free survival

## Abstract

The pretreatment prognostic nutritional index (PNI) is an indicator of nutritional and immune status, and has potential use as a predictor of survival in cancer patients. Several retrospective studies have used the PNI to predict the outcome of lung cancer patients receiving different immune checkpoint inhibitors (ICIs), but the results have been inconsistent. The objective of our study is to assess the relationship of pretreatment PNI with survival outcomes in lung cancer patients who received ICI-based treatments by meta-analysis.

**Methods:**

We searched the EMBASE, PubMed, Cochrane Library, American Society of Clinical Oncology, and European Society of Medical Oncology databases to identify studies that reported overall survival (OS) or progression-free survival (PFS) in eligible patients. Eight studies were eligible based on predefined inclusion and exclusion criteria. Data and pooled indicators were extracted from these studies. Meta-analysis was used to analyze hazard ratios (HRs) and 95% confidence intervals (CIs) for OS and/or PFS and the prognostic value of pretreatment PNI. We completed the registration of the research protocol (Registration number: INPLASY202240087, DOI number: 10.37766/inplasy2022.4.0087).

**Results:**

We analyzed data from 8 eligible studies (831 patients). Meta-analysis showed that relative to patients with low pretreatment PNI, those with a high pretreatment PNI had better OS (HR = 2.50, 95% CI = 1.44–4.33, *P* = .001) and better PFS (HR = 1.94, 95% CI = 1.56–2.42, *P* < .001). Sensitivity analysis indicated these results were robust. There was also no evidence of publication bias.

**Conclusion:**

Lung cancer patients receiving ICI-based treatments who had higher pretreatment PNI had better OS and PFS.

## 1. Introduction

Lung cancer is responsible for the greatest number of cancer deaths worldwide, and has a high incidence and mortality rate in men and women.^[1]^ The introduction of immunotherapy for lung cancer, especially immune checkpoint inhibitors (ICIs), has greatly increased the survival times of patients with lung cancer.^[2]^ At present, ICIs can be used as first-line treatments for advanced non-small cell lung cancer (NSCLC), the extensive stage of small cell lung cancer (SCLC), and as neoadjuvant or adjuvant therapy for NSCLC.^[3]^ Although ICIs have good efficacy and are well-tolerated, some patients do not achieve good treatment response.^[4]^ It is therefore important to use clinically accessible biomarkers or other methods to identify patients who are most likely to have long-term favorable responses to ICIs.

Many studies have examined the use of simple biomarkers from blood tests to determine the prognoses of patients with a variety of cancers. Thus, biomarkers such as the prognostic nutritional index (PNI),^[5]^ neutrophil-to-lymphocyte ratio, platelet-to-lymphocyte ratio, red blood cell distribution width, lung immune prognostic index, and lactate dehydrogenase, have been used to assess the immune status, nutritional status, or inflammatory status of cancer patients.^[6]^ The PNI is a particularly useful biomarker because it indicates the nutritional and immunological status of cancer patients. In 1980, researchers in the U.S. introduced the PNI as a nutritional indicator to predict risk from gastrointestinal surgery.^[7]^ In 1984, researchers in Japan simplified the algorithm for calculating PNI as: (10 × albumin [g/dL]) + (0.005 × lymphocytes [cells/µL]).^[8]^ More recent studies have extended the PNI to predict prognosis in additional groups of patients, such as those with gastrointestinal malignancies, gynecological tumors, and lung cancer.^[9–13]^ In particular, numerous retrospective clinical studies have examined the value of pretreatment PNI in assessing the prognosis of lung cancer patients receiving ICI therapies.^[14–21]^

The research problem of this meta-analysis was to determine the prognostic value of pretreatment PNI in lung cancer patients receiving ICI-based treatment by investigating the association between pretreatment PNI and prognosis of lung cancer patients receiving ICI-based treatment. Our objective was to find out whether there was an association between pretreatment PNI and prognosis of lung cancer patients receiving ICI-based therapy, and if so, how pretreatment PNI affected OS and PFS.

## 2. Materials and Methods

This study adhered to the PRISMA guidelines and developed exclusion criteria based on the PICOS model. The registration number is INPLASY202240087 and the DOI number is 10.37766/inplasy2022.4.0087.

### 2.1. Study search

Two investigators (SYF and CW) independently searched EMBASE, PubMed, Cochrane Library, American Society of Clinical Oncology, and European Society of Medical Oncology using the following terms: PNI; immunotherapy; immune checkpoint inhibitor; programmed death ligand-1 inhibitor; programmed death-1 inhibitor. The results included published and unpublished studies. References in these documents were also examined to identify additional relevant studies. If there was disagreement about the results of the search or study quality, a third member of the team (GXL) reviewed the results until the 3 investigators reached a consensus.

### 2.2. Study selection

#### 2.2.1. Inclusion criteria.

All included studies: were clinical trials that examined patients with lung cancer based on cytology or histology; examined patients who received treatments that included ICIs; used the PNI as a prognostic indicator of outcome (progression-free survival [PFS] or overall survival [OS]) and provided hazard ratios (HRs) with 95% confidence intervals (CIs) for these metrics; measured the PNI prior to administration of a regimen containing an ICI.

#### 2.2.2. Exclusion criteria.

All included studies: were excluded if they only calculated HRs using univariate analysis; were reviews, comments, letters, expert opinions, summary of meetings or reports; have no adequate and accurate definition of PNI and its pre- and/or post-treatment cutoff values; were repeated study data.

### 2.3. Data extraction

**Figure 1.**
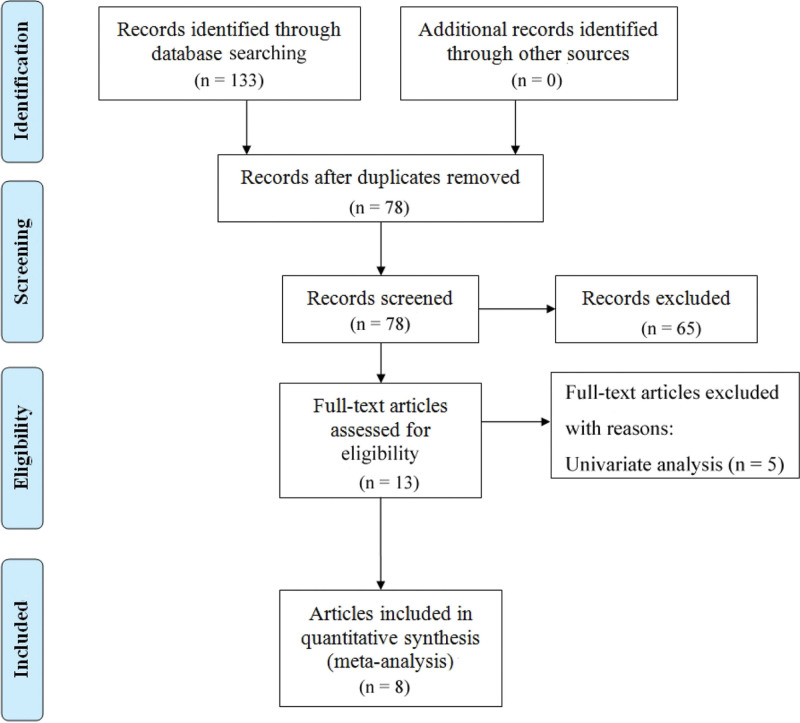
Identification, screening, assessment of eligibility, and inclusion of studies.

A total of 133 potentially eligible studies were identified by database searching, and 8 studies were considered eligible for inclusion (Fig. 1). Two investigators separately performed data extraction from the 8 included studies, and recorded the following information in an Excel spreadsheet: name of the first author, date of publication, time period when patients were enrolled, country where the study was conducted, study design, sample size, patent gender, follow-up time, type of cancer, treatment regimen, pretreatment PNI cutoff value, survival outcome (PFS or OS), and HR with 95% CI (Table 1).

**Table 1:** Characteristics of the included studies.*

First author, yr	Country	Duration	Sample size, n	Age, yrs	Gender, F/M	Follow-up, months	Cancer type	Treatment	PNI cutoff	Outcome	NOS
Shoji et al, 2019	Japan	2015–2019	102	range: 42–86	29/73	median: 7.37	NSCLC	ICI	45.5	OS,PFS	8
Peng et al, 2020	China	2017–2019	102	median: 62	15/87	NR	NSCLC	ICI	45	OS,PFS	7
Liu et al, 2021	China	2018–2019	123	mean: 59.9	25/98	NR	NSCLC	ICI + Chemo	46.05	OS,PFS	7
Qi et al, 2021	China	NR	53	NR	19/34	median: 17.1	SCLC	ICI + Chemo	48	OS	8
Shi et al, 2021-1	China	2015–2020	32	NR	NR	median: 12.9	NSCLC	ICI	45	OS,PFS	8
Shi et al, 2021-2	China	2015–2020	71	NR	NR	median: 12.9	NSCLC	ICI + Chemo	45	OS,PFS	8
Zaitsu et al, 2021	Japan	2016–2020	73	mean: 70.9	21/52	NR	LC	ICI	43	OS,PFS	7
Shijubou et al, 2022	Japan	2017–2019	38	median: 75	8/30	NR	NSCLC	ICI	40	PFS	7
Tanaka et al, 2022	Japan	2018–2020	237	median: 69	50/187	median: 11.7	NSCLC	ICI + Chemo	40.35	OS,PFS	8

* All studies were retrospective.

NR: not reported; F, female; M, male; LC: lung cancer; NSCLC: no-small cell lung cancer; SCLC: small cell lung cancer; OS: overall survival; PFS: progression-free survival; ICI: immune checkpoint inhibitor; NOS: Newcastle-Ottawa Scale; Chemo: chemotherapy.

Qi, W.X., et al, *Assessment of systematic inflammatory and nutritional indexes in extensive-stage small-cell lung cancer treated with first-line chemotherapy and atezolizumab.* Cancer Immunol Immunother, 2021. **70**(11): p. 3199-3206.

### 2.4. Assessment of study quality

The Newcastle-Ottawa Scale (NOS) was used to assess study quality^[22]^ (Table S1, Supplemental Digital Content, http://links.lww.com/MD/H630). In this scale, a score less than 5 indicated “low quality,” a score between 5 and 7 indicated “medium quality,” and a score greater than 7 indicated “high quality.”

### 2.5. Meta-analysis

STATA version 16.0 was used for statistical analysis. The primary endpoints were PFS and OS. HRs and 95% CIs were used to evaluate the relationship between pretreatment PNI and survival outcomes. A HR with 95% CI in a pooled analysis of survival outcome was considered significant if the *P* value was below .05. The chi-square test and the I^2^ statistic were used to evaluate statistical heterogeneity. When there was significant heterogeneity (*I^2^* > 50%), a random-effects model was used; when there was low heterogeneity (*I^2^* < 25%) or moderate heterogeneity (25% < *I^2^* < 50%) a fixed-effects model was used. Begg’s test and Egger’s test were used to assess publication bias.^[23,24]^ Sensitivity analysis was used to assess the robustness of the pooled HR values.

## 3. Results

### 3.1. Eligible studies

Our search of multiple databases led to the identification of 133 potentially eligible studies. Application of the predefined inclusion and exclusion criteria led to identification of 8 eligible studies (Fig. 1, Table 1). These 8 studies examined 831 patients with lung cancer who received ICI-based therapy, and all of them examined patients from East Asia (China and Japan). Six studies examined the association of pretreatment PNI with OS and PFS,^[14,15,17,19–21]^ 1 study only examined the relationship of pretreatment PNI with PFS,^[18]^ and 1 study only examined the relationship of pretreatment PNI with OS.^[16]^ One publications^[15]^ contained more precise groupings, so we separated the data from this article and recorded it as “Shi 2021-1” and “Shi 2021-2.” All 8 studies were retrospective and all had NOS scores of 7 or 8, indicating they were “high quality” studies.

### 3.2. Prognostic value of PNI for OS

**Figure 2.**
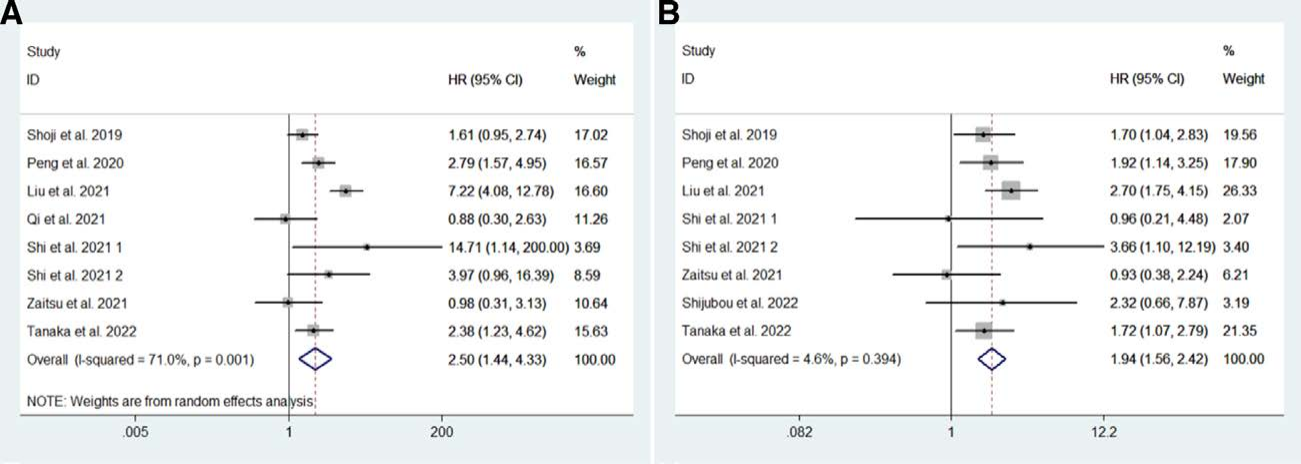
Meta-analysis of the impact of pretreatment PNI on overall survival (A) and progression-free survival (B). PNI = prognostic nutritional index.

Seven studies (793 patients) examined the relationship of pretreatment PNI with OS (Fig. 2A; (14-17, 19-21). These studies had high heterogeneity (*I^2^* = 71%, *P* = .001), so we used a random effects model for analysis. The results showed that patients with high pretreatment PNI had significantly better OS than patients with low PNI (HR = 2.50, 95% CI = 1.44–4.33, *P* = .001). We also performed subgroup analysis (Table S2, Supplemental Digital Content, http://links.lww.com/MD/H631) to determine the effect of country (China or Japan), sample size (n > 100 or n < 100), PNI cutoff value (PNI > 45 or PNI ≤ 45), treatment modality (ICI or ICI + chemotherapy), and NOS score (7 or 8). The results showed that high pretreatment PNI predicted better OS, regardless of country, sample size, treatment regimen, and NOS score. Subgroup analysis also suggested that it was more reasonable to use a PNI cutoff value of 45 or less.

### 3.3. Prognostic value of PNI for PFS

Seven studies (778 patients) examined the relationship of pretreatment PNI with PFS (Fig. 2B) (14, 15, 17, 19-21, 25). These studies had low heterogeneity (*I^2^* = 4.6%, *P* = .0394), so a fixed-effects model was used for analysis. The results showed that patients with high pretreatment PNI had significantly better PFS than patients with low PNI (HR = 1.94, 95% CI = 1.56–2.42, *P* < .001). Subgroup analysis was performed as previously, and indicated that high pretreatment PNI predicted better PFS, regardless country, sample size, treatment regimen, and NOS score (Table S3, Supplemental Digital Content, http://links.lww.com/MD/H632).

### 3.4. Publication bias and sensitivity analysis

**Figure 3.**
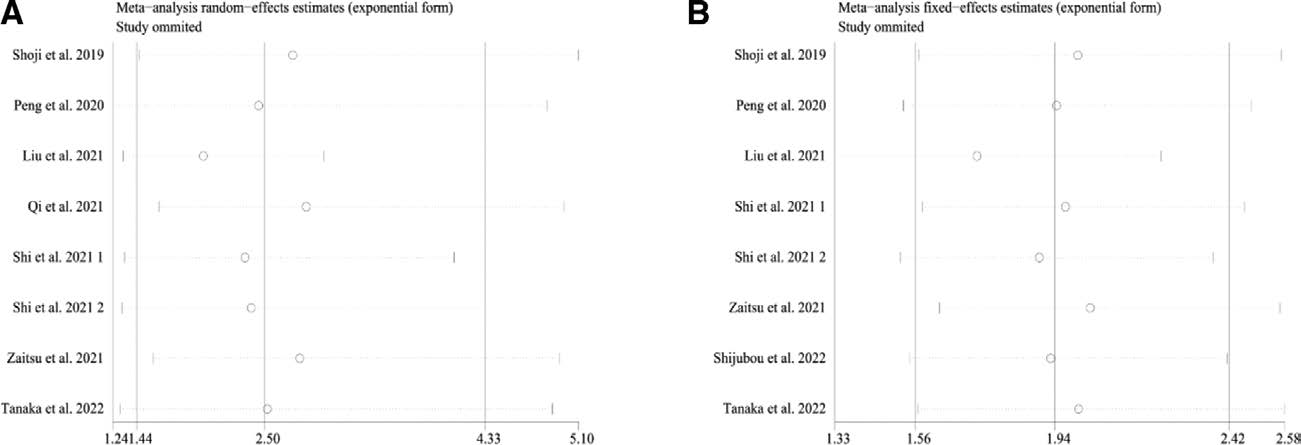
Sensitivity analyses of HR for OS (A) and PFS (B). HR = hazard ratios, OS = overall survival, PFS = progression-free survival.

Measurements using Egger’s test (*P* = .884 for OS, *P* = .549 for PFS) and Begg’s test (*P* = .536 for OS, *P* = .536 for PFS) indicated no evidence for significant publication bias. We also performed sensitivity analyses to validate the robustness of the pooled results for OS (Fig. 3A) and PFS (Fig. 3B). The results showed no significant change in the pooled HR for OS or PFS, indicating the pooled results were robust.

## 4. Discussion

Our major finding is that the pretreatment PNI is a reliable indicator of prognosis in lung cancer patients receiving ICI-based therapy. In particular, patients with a high pretreatment PNI had significantly better PFS and OS than those with a low pretreatment PNI. According to the results of our meta-analysis, survival time and progression-free time were shorter in the low pretreatment PNI group. The low pretreatment PNI group have a 150% increased risk of death and a 94% increased risk of disease progression than the high pretreatment PNI group. Since the introduction of the PNI in 1980, this index has been to estimate prognosis in patients with various diseases, and also in patients who received surgical treatment,^[25]^ radiotherapy,^[26]^ and drug therapy. Measurement of the PNI is simple and economical, and the results are applicable in diverse clinical fields. Several studies have demonstrated the usefulness of the PNI in predicting the prognosis of cancer patients.^[27,28]^ More specifically, the PNI is useful in predicting the prognosis of lung cancer patients, in that a high PNI was associated with better prognosis.^[9]^ Other studies examined use of the PNI to predict outcome in cancer patients receiving an ICI-based therapy and concluded that a high PNI predicted a better outcome.^[29]^

We identified 8 retrospective studies, all published since 2019, that examined use of the pretreatment PNI in predicting the outcome of lung cancer patients receiving ICI-based therapies. However, the conclusions of these studies were inconsistent. We therefore conducted a meta-analysis of these recent studies. All of these studies examined patients with lung cancer who received treatments that included ICIs, measured the pretreatment PNI, assessed PFS and/or OS, and provided HRs and 95% CIs for the outcome metrics. Our study is the only meta-analysis to focus on this topic. Seven^[14–17,19–21]^ of the studies in our included literature included data on patient OS. Four^[14,15,17,20]^ of these studies showed that pretreatment PNI was associated with patient OS and that a relatively high pretreatment PNI represented a better OS for patients, however the other 4 studies^[16,17,19,21]^ showed no statistical association between pretreatment PNI and patient OS. The heterogeneity of studies was relatively high when the 8 data sets from these 7 papers were analyzed in a pooled manner. We attributed the source of heterogeneity to differences in treatment regimens and differences in lung cancer type included in each study. Four data sets were treated with ICIs alone, however, the other 4 sets were treated with ICIs plus chemotherapy (Table 1). So we can infer that there may be a difference in the staging of lung cancer, it is an important source of heterogeneity. Five studies^[14,15,17,19,20]^ were conducted on NSCLC alone, 1 study^[16]^ was conducted on SCLC, and 1 study^[21]^ did not distinguish between NSCLC and SCLC. It is known that the OS of SCLC is much worse than that of NSCLC. So we think lung cancer type is also an important source of heterogeneity. Seven of the studies in our included literature included data on patient PFS. The low heterogeneity of our pooled results for PFS may be due to the relatively small differences in the characteristics of those patients included in the 8 data sets included in these 7 studies, with non-NSCLC cases accounting for a smaller number.

Clinicians have long used the PNI, which is a function of serum albumin and lymphocyte count (10 × Alb [g/dL] + 0.005 × lymphocytes [cells/µL]), as a prognostic indicator because it considers nutritional status and immune status. When we discuss the mechanisms involved in PNI affecting immunotherapy, we think in terms of 2 separate perspectives, albumin and lymphocytes.

Albumin accounts for about 55% of total plasma protein, and normal levels are about 35 to 45 g/L. Serum albumin is an indicator of nutritional status, it also helps to maintain intravascular osmotic pressure,^[30,31]^. A healthy liver synthesizes about 14 g of albumin per day, and albumin levels are generally lower in patients with hepatic dysfunction. The serum albumin level is also lower in patients with malignant tumors, severe tuberculosis, malnutrition, accumulation of thoracoabdominal fluid, kidney disease, and hypoalbuminemia. A low serum albumin level is therefore an independent indicator of poor prognosis in these patients with diverse conditions. Malnutrition is common in cancer patients, this is mainly due to the physical and metabolic effects of cancer and the side effects of anti-cancer treatments^[32,33]^, so hypoproteinemia is common in cancer patients. Epidemiological studies reported that a low pretreatment albumin level was associated with poor outcome in lung cancer patients^[34]^. At the cellular level, hypoalbuminemia may lead to impaired immune cell function, thereby allowing tumor progression^[35]^. Patients with tumor progression and poor nutritional status have poor prognoses. Another important consideration is that cytokines, such as interleukin-1, interleukin-6, and tumor necrosis factor-α, inhibit the synthesis of albumin^[30]^, and the levels of these cytokines are increased in patients with malignant tumors^[36,37]^. Albumin also functions in the scavenging of oxygen free radicals, a hallmark of inflammation^[31]^. Inflammation plays an important role in the development, progression, and metastasis of malignant tumors^[38]^. In patients targeted for immunotherapy, hypoproteinemia can lead to physiological dysfunction including loss of drug efficacy^[39]^, and it has been shown that shortened OS in individuals with higher pembrolizumab clearance is associated with increased cancer cachexia and protein turnover^[40]^. Talvas et al illustrates that the proper process of activation and function of cytotoxic T cells and memory cells requires adequate protein intake and^[41]^, in particular,arginine has a positive effect on the activity of the immune system^[42]^. Tumor interstitial fluid and the tryptophan and cystine consumed in the circulation of cancer patients, among others, play an important role in activating the immune response of the immune system^[43]^. Hypoproteinemia restricts the availability of these amino acids and may deprive immune cells of essential nutrients, thereby having the adverse effect of suppressing anti-cancer immunity and impeding the anti-cancer activity of ICIs. These many different functions of albumin explain the association of hypoalbuminemia with poor prognosis in patients with malignancies.

Lymphocytes function in immune recognition, and are classified as B cells, T cells and natural killer cells. All 3 types of lymphocytes have anti-tumor effects.^[44–49]^ T cells account for about 70 to 80% of all peripheral blood lymphocytes, and their anti-tumor effects is an area of active research. In particular, at the onset of tumorigenesis, T cells are activated, migrate to the lesion, and attack tumor cells.^[49]^ Previous studies of patients with breast cancer and lung cancer reported negative associations of peripheral blood T-cell count and circulating cancer cell load.^[50,51]^ More recent studies demonstrated that a low peripheral blood lymphocyte count was associated with lung cancer invasion and recurrence, and was an independent predictor of poor prognosis in these patients.^[52,53]^ Other studies also reported a relationship of cancer progression with low lymphocyte count in patients with pancreatic, esophageal, and kidney cancers.^[54–56]^ This suggests that a decreased peripheral blood lymphocyte count may reflect an impairment of the body’s anti-cancer response. Previous studies showed that immune checkpoints, such as programmed cell death protein 1 and cytotoxic T-lymphocyte-associated protein 4, down-regulate T-cell function, allowing immune evasion of malignant cells.^[57]^ Thus, a low serum albumin and a low lymphocyte count have many adverse effects, and this explains why the pretreatment PNI is such a reliable prognostic indicator for lung cancer patients receiving ICI-based treatment.

Although the exact mechanism by which PNI affects ICIs has not been fully cleared, the discussion above may suggest that a relatively high PNI at baseline status may reflect the patient’s original nutritional status and better immune system response. It may also reflect tumor differentiation in each individual, with patients with high pretreatment PNI having relatively less aggressive tumors. Therefore, such patients tend to have better OS and PFS. Many studies examined the use of PNI to predict disease outcome, and most of them concluded that a high pretreatment PNI was associated with a better prognosis.^[9,10,25–28,58–61]^ Studies have also looked at the relationship between PNI and immunotherapy and have found PNI to be an independent factor in the prognosis of immunotherapy patients.^[29,62]^ Compared to other indicators, PNI is more suitable for the prediction of survival in the elderly and in patients with malignancies because it considers nutritional status and immunological status. These 2 groups of patients often have poorer nutritional status due to chemotherapy or receipt of surgery. Moreover, tumor progression is also associated with a collapse of the body’s immune system. Sociologically speaking, patients with low pretreatment PNI often have poorer economic status, have less nutritious diets, receive worse medical care, and experience poor living conditions. These many factors related to lifestyle reduce the PNI and also adversely affect the immune system and patient prognosis. From our clinician’s perspective, with the current explosion of immunotherapy, we need to think about new models of managing patients, that is, the alignment of nutritional intake and immunotherapy. There have been many studies showing that diet can interfere with the efficacy of ICIs and thus have an impact on prognosis. In addition to regulating the function of immune cells through the regulation of metabolites such as amino acids, fatty acids and nucleotides in the body, diet can also influence the efficacy of ICIs by affecting the gut microecology.^[41,43,63,64]^

Although our meta-analysis confirmed the PNI was a reliable prognostic indicator, clinicians should use caution when using a single indicator to predict patient prognosis. Because the clinical studies we examined have limitations, like all clinical studies, we suggest that future efforts consider development of an algorithm that considers the PNI along with several additional indicators, such as the neutrophil-to-lymphocyte ratio, red blood cell distribution width, platelet-to-lymphocyte ratio, lung immune prognostic index, and lactate dehydrogenase to more accurately assess patient prognosis. Use of such an algorithm may allow clinicians to focus on the specific nutritional, immunological, or other specific problems in patients with malignant tumors.

Our study has some limitations. All of the 8 studies we examined enrolled patients from China and Japan, so the results may only be relevant to East Asian populations. All 8 studies were retrospective, and this may have led to bias. The sample size of our meta-analysis was also small, in that we only examined 8 studies with 831 patients, and these studies had significant heterogeneity regarding treatment regimen, lung cancer pathology, PNI cutoff, and patient age distribution. Finally, the 8 studies we examined did not provide sufficient data on drugs and dosing regimens, so we were unable to consider these factors in our analysis.

## 5. Conclusion

Our meta-analysis indicated that the pretreatment PNI of lung cancer patients was a reliable indicator of patient outcome. More specifically, patients receiving ICIs for lung cancer who had a higher pretreatment PNI had better OS and PFS.

## Author contributions

**Conceptualization:** Mingbo Tang.

**Data curation:** Yifeng Shao, Wei Cao, Xinliang Gao.

**Formal analysis:** Yifeng Shao.

**Investigation:** Yifeng Shao, Wei Cao, Xinliang Gao.

**Methodology:** Mingbo Tang.

**Project administration:** Mingbo Tang.

**Resources:** Wei Cao, Xinliang Gao.

**Software:** Yifeng Shao.

**Supervision:** Wei Liu.

**Validation:** Dongshan Zhu.

**Writing – original draft:** Yifeng Shao.

**Writing – review & editing:** Wei Cao.

## Supplementary Material


