# Supplementary material for: Pretreatment “prognostic nutritional index” as an indicator of outcome in lung cancer patients receiving ICI-based treatment: Systematic review and meta-analysis
Source: Medicine (Baltimore). 2022 Oct 28;101(43):e31113. doi: 10.1097/MD.0000000000031113 (PMC9622676; doi:10.1097/MD.0000000000031113)
Supplement: Supplementary file 1 [file medi-101-e31113-s001.pdf]

Supplementary Table 1. Quality assessment (Newcastle-Ottawa Scale) of the included studies.

	Selection				Comparability	Outcome	
	1	2	3	4	5	6	7
Shoji et al, 2019	★	★	★	★	★★	★	★
Peng et al, 2020	★	★	★	★	★★	★	-
Liu et al, 2021	★	★	★	★	★★	★	-
Qi et al, 2021	★	★	★	★	★★	★	★
Shi et al, 2021-1	★	★	★	★	★★	★	★
Shi et al, 2021-2	★	★	★	★	★★	★	★
Zaitsu et al, 2021	★	★	★	★	★★	★	-
Shijubou et al, 2022	★	★	★	★	★★	★	-
Tanaka et al, 2022	★	★	★	★	★★	★	★

1, Representativeness of the exposed cohort; 2, Selection of the non-exposed cohort; 3, Ascertainment of exposure; 4, Outcome of interest not present at start of study; 5, Comparability of cohorts on the basis of the design or analysis; 6, Assessment of outcome; 7, Follow-up long enough for outcomes to occur; 8, Adequacy of follow up of cohorts.
