# Supplementary material for: Pretreatment “prognostic nutritional index” as an indicator of outcome in lung cancer patients receiving ICI-based treatment: Systematic review and meta-analysis
Source: Medicine (Baltimore). 2022 Oct 28;101(43):e31113. doi: 10.1097/MD.0000000000031113 (PMC9622676; doi:10.1097/MD.0000000000031113)
Supplement: Supplementary file 2 [file medi-101-e31113-s002.pdf]

Supplementary Table 2. Subgroup analyses of the association between pretreatment PNI and OS.

Subgroup	Studies, n	HR, 95% CI	P	Heterogeneity	
				I^2, %	P_H
Country					
China	5	3.45 (1.55–7.71)	0.002	71.2	0.008
Japan	3	1.74 (1.18–2.57)	0.005	0	0.388
Sample size, n					
>100	4	2.96 (1.55–5.67)	0.001	79.9	0.002
<100	4	1.87 (0.67–5.21)	0.229	51.7	0.102
PNI cut-off					
>45	3	2.29 (0.68–7.72)	0.181	89.5	<0.001
≤45	5	2.51 (1.71–3.69)	<0.001	18.2	0.299
Treatment					
ICI	4	2.00 (1.39–2.88)	<0.001	47.5	0.127
ICI+Chemo	4	2.91 (1.17–7.22)	0.021	78	0.003
NOS					
7	3	3.00 (1.13–7.99)	0.028	82.2	0.004
8	5	1.89 (1.30–2.73)	0.001	35.1	0.187

Abbreviations: ICI: immune checkpoint inhibitor; NOS: Newcastle- Ottawa Scale; OS: overall survival.
