# Supplementary material for: Pretreatment “prognostic nutritional index” as an indicator of outcome in lung cancer patients receiving ICI-based treatment: Systematic review and meta-analysis
Source: Medicine (Baltimore). 2022 Oct 28;101(43):e31113. doi: 10.1097/MD.0000000000031113 (PMC9622676; doi:10.1097/MD.0000000000031113)
Supplement: Supplementary file 3 [file medi-101-e31113-s003.pdf]

Supplementary Table 3. Subgroup analyses of the association between pretreatment PNI and PFS.

Subgroup	Studies, n	HR, 95% CI	P	Heterogeneity	
				I^2 (%)	P_H
Country					
China	4	2.34 (1.71–3.20)	<0.001	0	0.428
Japan	4	1.62 (1.18–2.21)	0.002	0	0.587
Sample size					
>100	4	2.02 (1.59–2.57)	<0.001	0	0.451
<100	4	1.55 (0.88–2.76)	0.132	25.4	0.259
PNI cut-off					
>45	2	2.22 (1.60–3.08)	<0.001	46.1	0.173
⩽45	6	1.74 (1.28–2.35)	<0.001	0	0.505
Treatment					
ICI	5	1.64 (1.20–2.25)	0.002	0	0.606
ICI+Chemo	3	2.28 (1.67–3.11)	<0.001	20.4	0.285
NOS					
7	4	2.11 (1.56–2.86)	<0.001	35.8	0.198
8	4	1.76 (1.27–2.44)	0.001	0	0.563

Abbreviations: ICI: immune checkpoint inhibitor; NOS: Newcastle- Ottawa scale; PFS: progression-free survival.
